# Comparative Study of Sexual Function Between Post-Hypospadias Repair and Post-Circumcision Patients

**DOI:** 10.21315/mjms-12-2025-878

**Published:** 2026-04-30

**Authors:** Ong Xin Zen, Mohd Shahrulsalam Mohd Shah, Wan Azman Wan Sulaiman, Mohamed Ashraf Mohamed Daud

**Affiliations:** 1 Department of Surgery, School of Medical Sciences, Universiti Sains Malaysia, Health Campus, Kelantan, Malaysia; 2 Hospital Pakar Universiti Sains Malaysia, Universiti Sains Malaysia, Health Campus, Kelantan, Malaysia; 3 Reconstructive Sciences Unit, School of Medical Sciences, Universiti Sains Malaysia, Health Campus, Kelantan, Malaysia

**Keywords:** hypospadias, sexual behaviour, orgasm, masturbation, treatment outcome

## Abstract

**Background:**

Hypospadias is the most common congenital anomalies of the male genitalia. Literature on sexual function outcome post-hypospadias repair is scarce, especially in the Southeast Asian population. This study compares the sexual function between hypospadiacs post-repair and healthy circumcised adult males.

**Methods:**

This study was conducted in Hospital Pakar Universiti Sains Malaysia (HPUSM). Convenience sampling of patients who underwent hypospadias repair from 1995 to 2005 was done. Healthy circumcised males were recruited as controls. This questionnaire-based study utilised an adaptation of the Global Sexual Functioning (GSF) questionnaire, Sexual Satisfaction Score (SSS) questionnaire, and Penile Perception Score (PPS).

**Results:**

49 questionnaires were sent to eligible subjects after screening, while 80 questionnaires were sent to suitable controls. 26 subjects and 75 controls responded. The control group was older [34.43 (±5.789) vs. 28.92 (±7.255) years]. The control group reported better scores in sexual function, sexual satisfaction, and penile cosmesis. The GSF scores showed a low negative correlation with SSS (*r* = −0.384) when both groups were analysed together. A similar pattern was seen in the control but not the hypospadias group. The correlation between SSS and PPS differed between control and subject, indicating a discordant pattern across groups.

**Conclusion:**

This study revealed that post-hypospadias repair patients demonstrated poorer sexual function compared to circumcised controls. SSS is reliable in assessing sexual satisfaction in hypospadiacs. There is a low negative correlation between GSF score and SSS. Better penile cosmesis assessed using PPS translates to better sexual satisfaction in circumcised controls, but the result is discordant in hypospadiacs.

## Introduction

Hypospadias is the most common congenital anomaly in paediatric, with an incidence of 1 in every 200 to 300 male live births (1). Hypospadias is characterised by an ectopic ventrally positioned urethral meatus, ventral chordee, and excessive dorsal foreskin (2). The objective of hypospadias repair is to provide a straight penis with an external urethral opening at the tip that is functionally and cosmetically normal (3). There are numerous surgical techniques and modifications, making it difficult to reach a consensus on optimal outcomes.

Part of the problem is that well-designed prospectively controlled studies are limited (4). The American Academy of Pediatrics (AAP) recommends performing hypospadias repair between 6 and 12 months of age. The study by Ziada et al. (5) revealed a statistically significant difference in complication rates between patients in the older age groups and those within the recommended age group according to the AAP guidelines.

Many studies report high success rates following hypospadias correction, but some have claimed dissatisfaction with the appearance and size of their penises (6). There have been few studies on the long-term consequences of hypospadias treatment in childhood on sexual and psychosexual function (2). There were no such studies undertaken in the Southeast Asia region. The available studies are somewhat conflicting, with some writers concluding that post-hypospadias repair patients have significantly impaired sexual function, while others indicate similar psychosexual development to controls (2, 7–9).

The Global Sexual Functioning (GSF) questionnaire is a multidimensional instrument designed to evaluate various aspects of psychosexual functioning in both males and females. It is a single summary score derived from the sexual history form (SHF) by Nowinski and LoPiccolo (10). The GSF questionnaire addresses five domains of psychosexual functions (pertaining to males), namely, frequency of sexual activity, sexual desire, arousal, erectile function, and orgasmic function. It is a comprehensive assessment tool for psychosexual functioning, and it does not account for the dimension of sexual satisfaction.

There are several objective scorings for the assessment of sexual satisfaction. This study used the sexual satisfaction questionnaire developed by Agnieszka and Grażyna from Poland. It consists of 10 questions chosen from 10 items derived from an initial pool of 35 questions. Questions with less discriminatory power were omitted (11). This instrument was adopted in the present study as the Sexual Satisfaction Score (SSS) due to its simplicity and concise structure.

The appearance of the penis contributes significantly to male sexual confidence. A limited number of studies have demonstrated the impact of penile cosmesis on sexual outcomes and psychosexual functioning (12, 13). Many methods have been applied in the assessment of penile appearance in the past. Among them, the Penile Perception Score (PPS) stood out as a simple and objective assessment providing consistent results across different observers (14, 15). Therefore, this present study decided to incorporate this instrument as the primary assessment tool for penile appearance.

Most previous studies have compared post-hypospadias patients with a normal control group. However, the characteristics of the control group were not specified, specifically, whether the control group had undergone any form of surgery to the penis, for example, circumcision. A circumcised penis was selected as the control group to provide a more appropriate comparison with post-hypospadias repair, given that both conditions involve prior surgical intervention. A previous study has shown that circumcision is not associated with sexual dysfunction (16). Therefore, using circumcised males as a control is unlikely to bias the study outcomes.

## Methods

### Sample Size Calculation

The sample size was calculated using PS Software version 3.0 (Open access software, https://ps-power-and-sample-size-calculation.software.informer.com/download/) with the *t*-test option to compare two independent means. A Type I error probability of 5% and a statistical power of 80% were set, with a group allocation ratio of 3:1 (normal circumcision: post-hypospadias repair). Reference value for the sample size calculation was derived from the study conducted by Majstorovic et al. (2).[Table t1-08mjms3302_oa]

The largest required sample size was obtained for the variable orgasmic function. After accounting for an anticipated 10% dropout rate, the final sample size was adjusted to 75 normal circumcision patients and 25 post-hypospadias repair patients, resulting in a total of 100 participants.

### Strobe Chart

This study was conducted at Hospital Pakar Universiti Sains Malaysia (HPUSM). Patients who underwent hypospadias repair surgery at HPUSM between 1995 and 2005 were identified using convenience sampling. The pool consisted of patients who underwent the surgery under paediatric surgery, urology, and plastic surgery teams. A total of 132 patients (Figure 1) were identified. Surgical and patient details were obtained from the operation theatre records and the medical records unit. The inclusion criteria for subjects were age of more than 18 years old, having engaged in sexual intercourse, and having at least a Malaysian Certificate of Education (SPM) education level. Meanwhile, the exclusion criteria were being unable to be contacted via phone call and refusal to participate.

The control group was recruited via convenience sampling from Universiti Sains Malaysia (USM) staff, students, and their relatives. Recruitment ceased once the required sample size was reached. The inclusion criteria for controls were an age range between 18 and 50 years old, having engaged in sexual intercourse, having at least SPM education level, and having undergone circumcision. Meanwhile, the exclusion criteria were having undergone surgery on the penis other than circumcision and refusal to participate.

Subjects and controls were contacted by phone or in person. Link to the questionnaire (in the form of a Google Form) was sent to them via WhatsApp, email or QR code.

### Research Tools

This study employed a questionnaire-based design, utilising an adaptation of three instruments: the GSF questionnaire, the SSS questionnaire, and the PPS questionnaire. The questionnaires were translated into Malay to make them easier for the local participants to understand. The translated version was proofread by a language lecturer from the School of Languages, Literacies and Translation at USM. The final questionnaire is in dual language (both English and Malay languages, with the Malay language written in italic). The questionnaire was then created using Google Forms and distributed to participants.

### GSF Score

The GSF is a single summary score derived from the SHF by Nowinski and LoPiccolo. The SHF is a self-report instrument comprising 46 multiple-choice items, each with a variable number of response options and differing response scales (e.g., Item 1 has nine options, while Item 18 has six options). Each response option is numbered and accompanied by a corresponding verbal descriptor. The GSF score is based on 12 items. As some items are specific to males while others apply only to females, the items used to calculate the male and female scores differ slightly. To obtain the single summary score, selected SHF items are grouped into a 12-item scale representing either male or female GSF. The single summary score is derived using the following procedure:

- Converting the scores on each of the 12 items to a proportion of the maximum possible value (e.g., if on Item 1, where response options are numbered 1 to 9, the respondent answers “(4) twice a week,” this is converted to 4/9 = 0.44)
- Summing the 12 proportions
- Calculating the mean by dividing the total by the number of items that the respondent is deemed to have answered (usually 12)
- The resulting mean value, which is the GSF score, will be greater than 0 and less than 1. Lower scores indicate better functioning (10)

### Sexual Satisfaction Score

The SSS consisted of 10 questions. The scoring is based on a four-point Likert scale ranging from 1 to 4 points. Questions 1, 3, 5, 8, and 9 are coded the opposite. Higher scores indicate better sexual satisfaction (11).

### Penile Perception Score

The PPS is derived from scoring given to four components of the penis, namely, the meatus, glans, skin, and general appearance. Each component was scored using a four-point Likert scale ranging from very dissatisfied (1 point), dissatisfied (2 points), satisfied (3 points), to very satisfied (4 points). Higher scores indicate better perceived cosmesis (14–15).

### Data Collection

Following the completion of the Google Form by the participants, the information was combined into a Google Sheet and imported into the Statistical Package for the Social Sciences (SPSS) version 29 (IBM Corp., Armonk, NY, US) to get the final score for GSF, SSS, and PPS.

### Statistical Analysis

SPSS was used to analyse the data. The association between the GSF score, SSS, and PPS in the subject and control groups was examined using descriptive statistics. An independent sample *t*-test was used to compare the subject and control means under the presumption of a normal data distribution. The Mann-Whitney U test was used in case of abnormal data distribution. Categorical variables like educational level, surgical type, and complications were compared using the Fisher-Exact test. The value of normal data distribution is expressed as mean ± standard deviation (SD), while abnormal data distribution is expressed as median (interquartile range). Spearman’s correlation test was used to assess the degree of correlation between the three scores (GSF, SSS, and PPS) in both groups of participants. The strength of the correlation coefficient is considered to be high positive (negative) 0.70 to 0.90 (−0.70 to −0.90); moderate positive (negative) 0.50 to 0.70 (−0.50 to −0.70); low positive (negative) 0.30 to 0.50 (−0.30 to −0.50), and negligible correlation 0.00 to 0.30 (0.00 to −0.30) (17).

## Results

Hypospadias correction was performed on 132 individuals at HPUSM between 1995 and 2005. Eight of them had incomplete surgical records, while 53 had one-stage repairs and 71 had two-stage repairs.

For the total of 132 patients, 83 were excluded: 23 did not meet the inclusion criteria (no experience of sexual intercourse), while 60 of them were unable to be contacted either due to a change of contact number, residential address or deceased. A Google Form questionnaire was given to the remaining 49 eligible respondents, who consented to participate; 26 of them responded. Eighty people were given the questionnaire for the control group, and 75 of them responded.

### Demographics

Table 2 presents the general characteristics of the participants. The control group is generally older than the subject group [34.43 (5.789) years vs. 28.92 (7.255) years]. Both groups have similar education levels. The control group has a larger number of children compared to the subject (2.0 vs. 0.5). Occupation-wise, the subject group is more heterogeneous compared with the control group, whereby the majority in the control group works in the healthcare industry. The type of surgery and complication rate (complications that require surgical intervention, e.g., fistula, re-do surgery, and wound breakdown) of the post-hypospadias repair group are not statistically significant.

### GSF Score, SSS Score, and PPS

Table 3 compares the differences in GSF score, SSS, and PPS between the participants. There is a significant difference in scores between the post-hypospadias repair and circumcised control groups in all three scorings. The control group has a better sexual function, sexual satisfaction, and perceived penile cosmesis.

### Domains of GSF Score Analysis

Table 4 compares the individual domains that make up the GSF score. Control shows better function in terms of frequency of sexual activities, sexual desires, and arousal. However, a significant increase is seen only in the frequency of sexual activities (*P* < 0.001) and arousal (*P* = 0.002). Both groups show similar values in terms of erectile abilities. The post-hypospadias repair group shows better orgasmic abilities, but the increase was not significant (*P* = 0.479).

### Post-hypospadias Repair Subgroup Analysis

There are no significant differences in GSF score (*P* = 0.874), SSS (*P* = 0.957), and PPS (*P* = 1.000) between the two subgroups of subjects, which are those that underwent one-stage repair and two-stage repair.

The GSF score (*P* = 0.337), SSS (*P* = 0.616), and PPS (*P* = 0.978) of post-hypospadias repair groups with and without complications are not significantly different from one another.

### Correlation Between GSF Score, SSS, and PPS

Table 5 shows the correlations between GSF score, SSS, and PPS among both groups and individually. There is a negative low correlation between GSF score and SSS, while the correlation between GSF and PPS is negligible in the combined group. A similar result is demonstrated in the control group, but not the subject group. There is a positive low correlation between SSS and PPS in both the combined and control groups. However, when the same analysis is done in the subject group alone, the correlation becomes negatively low.

## Discussion

The study on sex-related topics remains a major challenge in Southeast Asian communities due to their conservative attitudes (18). Due to the delicate nature of this subject, the vast majority of healthy controls (*n* = 40) declined to participate in the present study. Asian cultures (18) and religious beliefs may have contributed to Southeast Asia’s conservative mindset (19).

Religion is also an important element that shapes one’s psychosexual development (20). Therefore, literature is scarce on sexual functions among the Southeast Asian population. Long-term psychosexual functions have been demonstrated to be significantly impacted by hypospadias correction (2, 5–9, 21). However, a direct comparison between the literature was not possible due to the heterogeneity of parameters measured and methods used. This is not surprising, as Nowinski and LoPiccolo’s SHF shows that sexual function is a broad term that encompasses numerous domains.

Regardless of the types of parameters measured, such studies are rare to begin with due to difficulties in approaching patients decades after the primary hypospadias repair (2). The present study encountered similar challenges when attempting to locate these patients, as most of the contact numbers obtained from the record unit were no longer active and therefore unreachable. Furthermore, the manual systems for health record keeping before the implementation of electronic health record systems proved to be a challenge for contact tracing. Some of the medical records had been disposed of in accordance with hospital policy, as they had remained inactive for more than seven years. These are the major factors leading to the high number of patients (*n* = 60) being excluded due to the lack of viable methods to contact them.

Despite the variety of methods used for assessment of psychosexual functions in post-hypospadias repair patients, the majority of the literature has concluded that hypospadias patients, despite surgical repair, will have poorer psychosexual function as compared to healthy controls (2, 5–9, 21). The study demonstrated similar findings, with significant differences observed in the GSF score, SSS, and PPS between hypospadias patients and the control group.

As mentioned previously, there is no standardised questionnaire for psychosexual function evaluation in post-hypospadias repair. The GSF was utilised in this study because it provides a comprehensive assessment across different domains of sexual function. In the evaluation of the five sexual domains, circumcised controls demonstrated a significantly higher frequency of sexual activity and improved arousal compared to the study group. There is no significant difference in erectile abilities between the two groups, which coincides with a few other studies that reported hypospadias patients have good erection ability (8, 12). Post-hypospadias repair patients showed a marginal increase in orgasmic ability scores, but the difference did not achieve statistical significance. This is a stark contrast to Majstorovic et al.’s (2) study, which demonstrated a better score in the control. This cannot be explained by the age factor alone, as a previous study showed a negligible association between age and orgasm rate (22).

Tack et al. (23) found that multiple surgeries are a risk factor for poorer psychosexual outcomes. In the present study’s subgroup analysis, however, no significant difference is noted between one-stage and two-stage repairs. Neither is there a significant difference between patients with complications that required surgery and those without. This might be explained by the relatively small number of participants in the subgroups, as was the case in Bubanj et al.’s (8) study.

In terms of cosmesis, the PPS is a commonly used tool for assessing penile appearance in post-hypospadias surgery patients. Many studies have attributed a particular surgical technique to a superior cosmesis outcome, such as Ververidis et al. (24), Oswald et al. (25), Shahid and Rahman (26), and Selvi et al. (27). Most of them found that the modified tabularised incised plate urethroplasty (Snodgrass) technique resulted in better cosmesis. Hamid et al.’s (28) study comparing Snodgrass and Mathieu’s procedure for primary (single-stage) hypospadias repair found that the Snodgrass repair is significantly faster, with a more natural cosmetic appearance of the meatus than Mathieu’s repair. As for the two-stage procedure for proximal hypospadias repair, a study demonstrates that the modified Bracka technique produces better cosmetic results with a far lower rate of complications (29).

Due to the incomplete records available, surgical techniques were not analysed in the present study. According to the available data, the Snodgrass technique was utilised for most one-stage hypospadias repairs, while the Bracka technique was employed for two-stage surgeries.

Subgroup analysis among hypospadiacs that underwent single versus two-stage procedures was underpowered and failed to show any significant differences between the two groups. Currently, there is a lack of literature exploring the association between hypospadias surgical repair techniques and sexual satisfaction.

A low negative correlation was found between GSF score and SSS when both groups’ data were analysed together. Similar results are only seen in the control group, but not in the subject group. Minimal association between GSF and PPS was observed in both separate and combined group analyses. The SSS and PPS have a low positive correlation when analysed in the combined group and in the control group; this correlation becomes low negative in the hypospadias group. This finding is contradictory to the common belief that a better penile cosmesis (higher PPS) translates to better sexual satisfaction (higher SSS). This discordance correlation has actually been described in prior literature, such as Singh et al. (7) and Aho et al. (30), who found that although hypospadiacs were markedly more dissatisfied with the result of the surgery, sexual life and success in life in general did not differ from those of circumcised patients.

Fertility-related literature in hypospadias patients is limited. Bracka found that 25% of 169 men who underwent childhood repair had oligospermia (31). Aho et al. (30) also reported that patients with hypospadias had fewer children, which is consistent with the present study’s findings that the control group had a higher number of children compared to hypospadias patients. This might simply be due to the age factor, as the control group is on average six years older than the hypospadias group (34.43 vs. 28.92 years old). Fertility issues such as diminished semen volume, sperm count, sperm concentration, motility, and morphology are more often seen in proximal hypospadias patients. A study by Punjani and Lamb (32) also pointed out that physical changes to the penis, whether primary or as a result of surgery, cause ejaculation issues, which creates a barrier for natural conception. A recently published cohort study by Lavoie et al. (33) regarding the prevalence of disorders of sex development (DSD) in patients with a history of both hypospadias and undescended testicles (UDT) found an increase in DSD diagnosis in proximal hypospadias patients with non-palpable UDTs. DSD is known to be associated with oligospermia or azoospermia, which might explain Bracka’s findings (34).

### Study Limitation

The limitation of the current study is that it does not have a sufficient sample in the hypospadias group to show a significant difference in terms of sexual function, sexual satisfaction, and penile perceptions among hypospadiacs that underwent a one-stage repair compared to a two-stage repair. The sample size is also insufficient to accurately compare the difference between hypospadiacs with complications which required surgery(-ies) and those without.

### Suggestion for Improvement

In future investigations, a larger sample of hypospadias patients can be recruited due to the ease of telecommunication as well as the improvement in electronic medical record systems. This can allow much valuable information regarding the patient, the type of surgery, and its complications to be traced accurately despite the passage of time. In the future, a sufficiently powered study may be able to describe the optimal method for repairing hypospadias that will produce the best results in terms of appearance and sexual function.

## Conclusion

This study concluded that post-hypospadias repair patients have poorer sexual functions compared to circumcised controls assessed using the GSF score. The SSS is a reliable questionnaire to assess sexual satisfaction in hypospadias patients. There is a low negative correlation between GSF score and SSS. Better penile cosmesis assessed using PPS translates to better sexual satisfaction in circumcised controls, but the result is discordant in hypospadiacs.

## Figures and Tables

**Figure 1:**
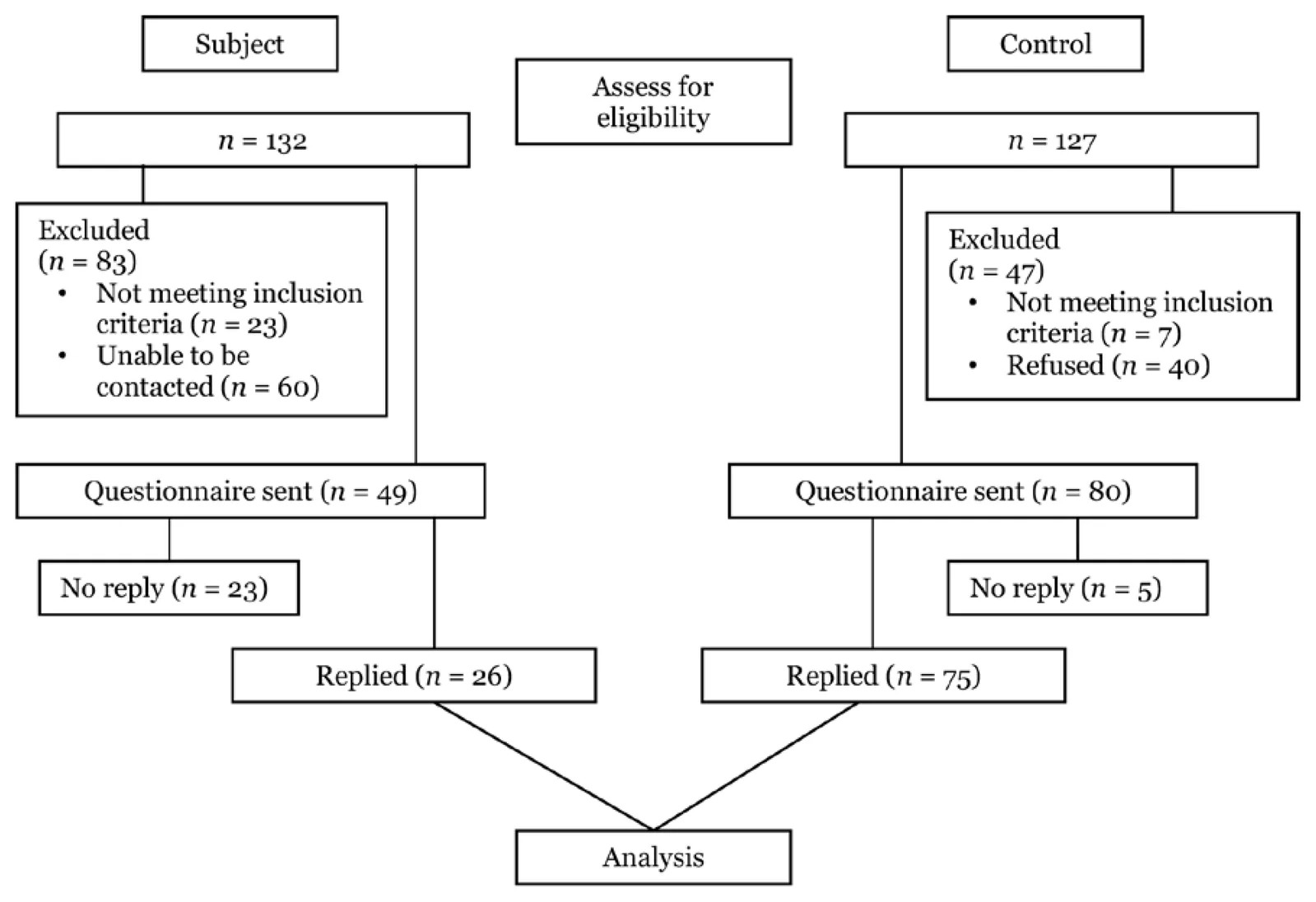
Strobe chart

**Table 1 t1-08mjms3302_oa:** Sample size calculation

Domain	Standard deviation (SD)	Expected difference	Calculate sample size	

			Normal circumcision	Hypospadias
Frequency of sexual activities	0.14	0.1	63	21
Sexual desire	0.25	0.2	51	17
Arousal	0.25	0.2	51	17
Erectile abilities	0.28	0.2	63	21
Orgasmic abilities	0.29	0.2	66	22
Sexual satisfaction	4.97	4.0	51	17

**Table 2:** Participant demographics and type of hypospadias surgery

Variable		Subject	Control	Statistical significance (*P* < 0.05)
Age (year)*		28.92 (7.255)	34.43 (5.789)	0.001^a^
Education level**		Degree	Degree	0.967^b^
No. of children***		0.5 (2)	2.0 (2)	0.033^c^

**Type of hypospadias surgery and complication rate**				

**Stages of surgery**		**One (** ***n*** ** = 10)**	**Two (** ***n*** ** = 16)**	**Statistical significance (** ***P*** ** < 0.05)**

Complications	Yes	4	7	
	No	6	9	1.0^b^
	Rate (%)	40	44	

* Mean (SD);

a Independent *t*-test;

** Mode;

b Fisher’s exact test;

*** Median (interquartile range);

c Mann-Whitney U test

**Table 3:** GSF score, SSS and PPS between the subject and control

Score	Subject	Control	Statistical significance (*P* < 0.05)
GSF score*	0.545 (0.237)	0.433 (0.119)	0.007**
SSS***	23.000 (3.072)	29.870 (3.811)	< 0.001****
PPS***	12.000 (2.191)	12.890 (2.442)	0.048****

* Median (interquartile range);

** Mann-Whitney U test;

*** Mean (SD);

**** Independent *t*-test

**Table 4:** Difference between domains in subject and control

Domain	Subject	Control	Statistical significance (*P* < 0.05)
Frequency of sexual activities* (GSF 1 and 2)	0.686 (0.269)	0.472 (0.175)	<0.001**
Sexual desires* (GSF 6 and 7)	0.671 (0.249)	0.662 (0.177)	0.870**
Arousal* (GSF 10 and 16)	0.539 (0.165)	0.410 (0.177)	0.002**
Erectile abilities*** (GSF 18, 19, and 22)	0.167 (0.220)	0.167 (0.170)	0.962****
Orgasmic abilities*** (GSF 23, 24, and 25)	0.317 (0.340)	0.400 (0.300)	0.479****

* Mean (SD);

** Independent *t*-test;

*** Median (interquartile range);

**** Mann-Whitney U test

**Table 5:** Correlations between GSF score, SSS, and PPS

Correlations*	Combined	Correlation coefficient	Control	Correlation coefficient	Subject	Correlation coefficient
GSF vs. SSS	< 0.001	−0.384	< 0.010	−0.431	0.184	0.269
GSF vs. PPS	0.008	−0.264	0.045	−0.233	0.401	−0.172
SSS vs. PPS	< 0.001	0.328	< 0.001	0.388	0.010	−0.496

* Spearman’s correlation
